# Reactivation of Latent Epstein-Barr Virus: A Comparison after Exposure to Gamma, Proton, Carbon, and Iron Radiation

**DOI:** 10.3390/ijms19102961

**Published:** 2018-09-28

**Authors:** Satish K. Mehta, David C. Bloom, Ianik Plante, Raymond Stowe, Alan H. Feiveson, Ashlie Renner, Adit Dhummakupt, Dhruv Markan, Ye Zhang, Honglu Wu, Blaire Scoles, Jeffrey I. Cohen, Brian Crucian, Duane L. Pierson

**Affiliations:** 1 KBRwyle, 2400 NASA Parkway, Houston, TX 77058, USA; ianik.plante-1@nasa.gov; 2 Department of Molecular Genetics & Microbiology, University of Florida, Gainesville, FL 32610, USA; dbloom@UFL.EDU (D.C.B.); adhummakupt@gmail.com (A.D.); blaire.scoles@gmail.com (B.S.); 3 Microgen Laboratoires, La Marque, TX 77568, USA; rpstowe@microgenlabs.com (R.S.); ashlin.renner@gmail.com (A.R.); 4 NASA Johnson Space Center, Houston, TX 77058, USA; alan.h.feiveson@nasa.gov (A.H.F.); honglu.wu-1@nasa.gov (H.W.); brian.crucian-1@nasa.gov (B.C.); duane.l.pierson@nasa.gov (D.L.P.); 5 Albert Einstein College of Medicine, Bronx, NY 10461, USA; dmarkan23@gmail.com; 6 NASA Kennedy Space Center, Cape Canaveral, FL 32899, USA; ye.zhang-1@nasa.gov; 7 Laboratory of Infectious Diseases, National Institutes of Health, Bethesda, MD 20892, USA; JCOHEN@niaid.nih.gov

**Keywords:** space radiation, Epstein‒Barr virus, virus reactivation

## Abstract

Among the many stressors astronauts are exposed to during spaceflight, cosmic radiation may lead to various serious health effects. Specifically, space radiation may contribute to decreased immunity, which has been documented in astronauts during short- and long-duration missions, as evidenced by several changes in cellular immunity and plasma cytokine levels. Reactivation of latent herpes viruses, either directly from radiation of latently infected cells and/or from perturbation of the immune system, may result in disease in astronauts. Epstein‒Barr virus (EBV) is one of the eight human herpes viruses known to infect more than 90% of human adults and persists for the life of the host without normally causing adverse effects. Reactivation of several latent viruses in astronauts is well documented, although the mechanism of reactivation is not well understood. We studied the effect of four different types of radiation, (1) ^137^Cs gamma rays, (2) 150-MeV protons, (3) 600 MeV/n carbon ions, and (4) 600 MeV/n iron ions on the activation of lytic gene transcription and of reactivation of EBV in a latently infected cell line (Akata) at doses of 0.1, 0.5, 1.0, and 2.0 Gy. The data showed that for all doses used in this study, lytic gene transcription was induced and median viral loads were significantly higher for all types of radiation than in corresponding control samples, with the increases detected as early as four days post-exposure and generally tapering off at later time points. The viability and size of EBV-infected Akata cells were highly variable and exhibited approximately the same trend in time for all radiation types at 0.1, 0.5, 1.0, and 2.0 Gy. This work shows that reactivation of viruses can occur due to the effect of different types of radiation on latently infected cells in the absence of changes or cytokines produced in the immune system. In general, gamma rays are more effective than protons, carbon ions, and iron ions in inducing latent virus reactivation, though these high-energy particles did induce more sustained and later reactivation of EBV lytic gene transcription. These findings also challenge the common relative biological effectiveness concept that is often used in radiobiology for other end points.

## 1. Introduction

### 1.1. Stressors in a Space Environment

In the space environment, astronauts are exposed to multiple stressors such as isolation, confinement, variable gravitational forces, increased radiation, psychosocial stressors, sleep deprivation, noise, physical exertion, deconditioning, and anxiety [1]. Among these stressors, the radiation environment in low-Earth orbit is composed of a complex mixture of galactic cosmic rays (GCR), particles of trapped belts, and secondary particles generated in both the spacecraft and Earth’s atmosphere [2]. Ions of all atomic numbers from hydrogen to uranium are present in GCR, the great majority being protons. Their energies cover a large spectrum extending from a few MeV/n to nearly 10^15^ MeV/n, peaking at ~1 GeV/n. On Earth, heavy ions are shielded by the Earth’s magnetosphere and atmosphere. In space, many highly charged (Z) and energy (HZE) particles, such as iron ions, are difficult to shield with existing methods. Furthermore, they may collide with nuclei of the atoms they encounter, fragmenting either the target and/or the projectile thereby producing several secondary particles. Therefore, space radiation is a concern for long-duration space missions, such as the three-year Mars mission that would lead to a whole-body dose of about 1 Sievert or more [3]. 

### 1.2. Reactivation of Latent Viruses

Immune system dysregulation, defined by altered leukocyte distribution, reduced T and NK cell function, and altered plasma cytokine levels, has been reported in astronauts during short- and long-duration spaceflight [4,5]. Reactivation of latent herpes viruses results as a consequence of decreased immunity in astronauts [6,7,8,9]. A central question is whether the detection of increased levels of viruses in humans during spaceflight is due to immunosuppression associated with travel in space or a direct effect of space environmental factors, such as radiation inducing reactivation of the virus from latency. For example, the primary infection by the varicella zoster virus (VZV) results in varicella (chickenpox), after which the virus becomes latent in ganglionic neurons along the entire neuraxis. When cellular immunity to VZV wanes with age and/or immunosuppression, VZV can reactivate to cause herpes zoster (shingles). The consequences of latent viral reactivation are important as shingles may be complicated by post-herpetic neuralgia, zoster paresis, cranial nerve palsies, myelitis, meningoencephalitis, vasculopathy, or ocular disorders [10]. Similarly, reactivation of Epstein‒Barr virus (EBV) may lead to lymphoproliferative disease and may play a role in Hodgkin’s lymphoma, peripheral B and T cell lymphomas, and nasopharyngeal carcinoma [11]. 

We have studied the effects of four types of radiation, ^137^Cs gamma, 150-MeV protons, 600 MeV/n carbon ions, and 600 MeV/n iron ions on the reactivation of EBV in an EBV-producer line (Akata) in vitro. This cell line was established from a Japanese patient with Burkitt’s lymphoma [12]. There are several reasons for the choice of radiation types. The ^137^Cs gamma ray is a common source of photons, which are often used in experiments as a reference for low linear energy transfer (LET). Protons are an obvious choice as they are the most important component of the galactic cosmic ray (GCR) spectra. The 150-MeV protons have a low LET and are considered to have a relative biological effectiveness (RBE) of 1.1 for several biological end points [13]. The RBE value of 1.1 for protons is based mostly on cell colony formation data, generally at higher, therapy-type dosages. Although it does not necessarily apply to other biological end points, it is an indication that protons and photons have similar biological effects at the same dose. The carbon ions are present in GCR and are also considered for hadron therapy. The iron ions are a relatively rare occurrence in the GCR, but they have high LET and high RBE for several biological end points [13]. Therefore, by using these ions, which cover a large part of the GCR spectra, the biological effects of representative particle types in space can be determined.

In this study, we found that a significant increase in EBV reactivation relative to controls is observed for all radiation types used. However, when equal doses (physical doses not corrected for the RBE) are compared with control, each radiation source induced viral reactivation differently. In general, gamma rays are more effective than protons, carbon ions, and iron ions in inducing latent virus reactivation. These findings challenge the common RBE concept that is often used in radiobiology for other end points [13]. This difference in viral reactivation may be explained by the differences in the radiation track structure. 

## 2. Results

**Figure 1:**
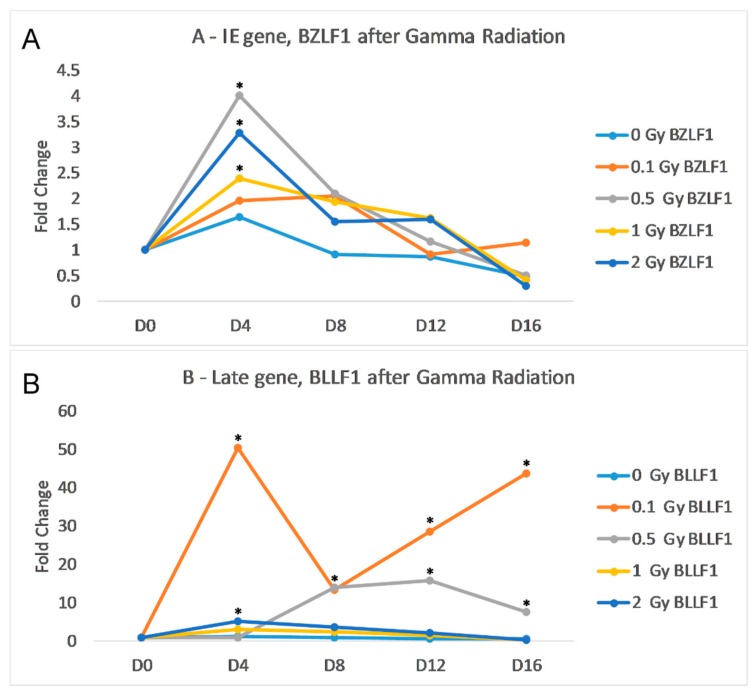
Effects of gamma radiation on Akata cells. Cells were radiated with 0.1 Gy (orange), 0.5 Gy (grey), 1 Gy (yellow) or 2 Gy (dark blue), then incubated for 16 days. The control flask (light blue) was not irradiated. (**A**) Fold change of IE gene BZLF1 transcripts normalized to endogenous control GAPDH over time relative to the control. (**B**) Fold change of L gene BLLF1 transcripts normalized to endogenous control GAPDH over time relative to the control. Statistical significance as compared to no radiation control and (two-tailed *t* test, * *p* < 0.05).

We sought to identify any increase in transcription of EBV lytic genes following treatment of Akata cells (that contain a latent EBV episome) with gamma irradiation (as described in the Materials and Methods sections). For these analyses we examined the RNA levels of two key EBV lytic genes, *BZLF* and *BLLF*. *BZLF1* is an immediate early *(IE)* gene that codes for *ZEBRA or Zta*, an important trans-activator that is critical for reactivation. *BLLF1* is a late *(L)* gene that codes for the envelope glycoprotein gp350; this protein complexes with gp220 to bind to the B-cell’s CD21. The results of the qRT-PCR indicate that gamma radiation induces transcription of both BZLF1 and BLLF peaking at day 4. post-gamma-radiation treatment (Figure 1). The irradiation dose of 0.1 Gy did not significantly increase the transcription of BZLF1; however, with 2.0 Gy of gamma radiation, there was a noticeable increase in both gene transcripts. The peak of BZLF1 transcription was at day 4 post-infection, but the increase was not statistically significant due to large standard deviation. At day 8, both 1.0 Gy and 2.0 Gy of gamma radiation resulted in significantly higher levels of BZLF1 (Figure 1A). 0.1 Gy of radiation caused a significant increase in BLLF1 from day 0 to day 16, while 0.5 Gy of radiation caused a significant increase at day 8 (Figure 1B). While gamma radiation increased the transcription in both gene classes, the amount of BLLF1 transcription was on average 3-fold less than BZLF1.

**Figure 2:**
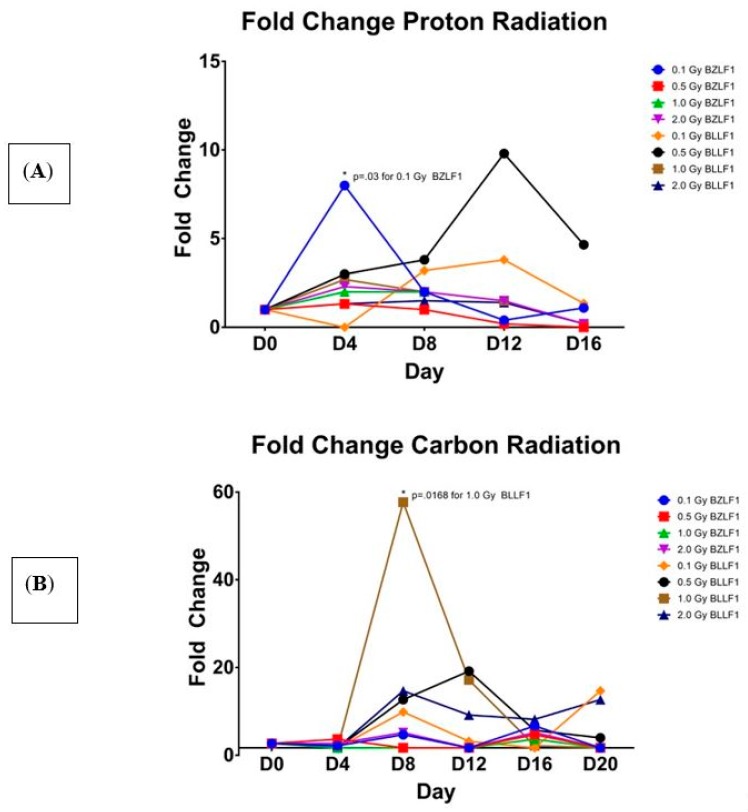

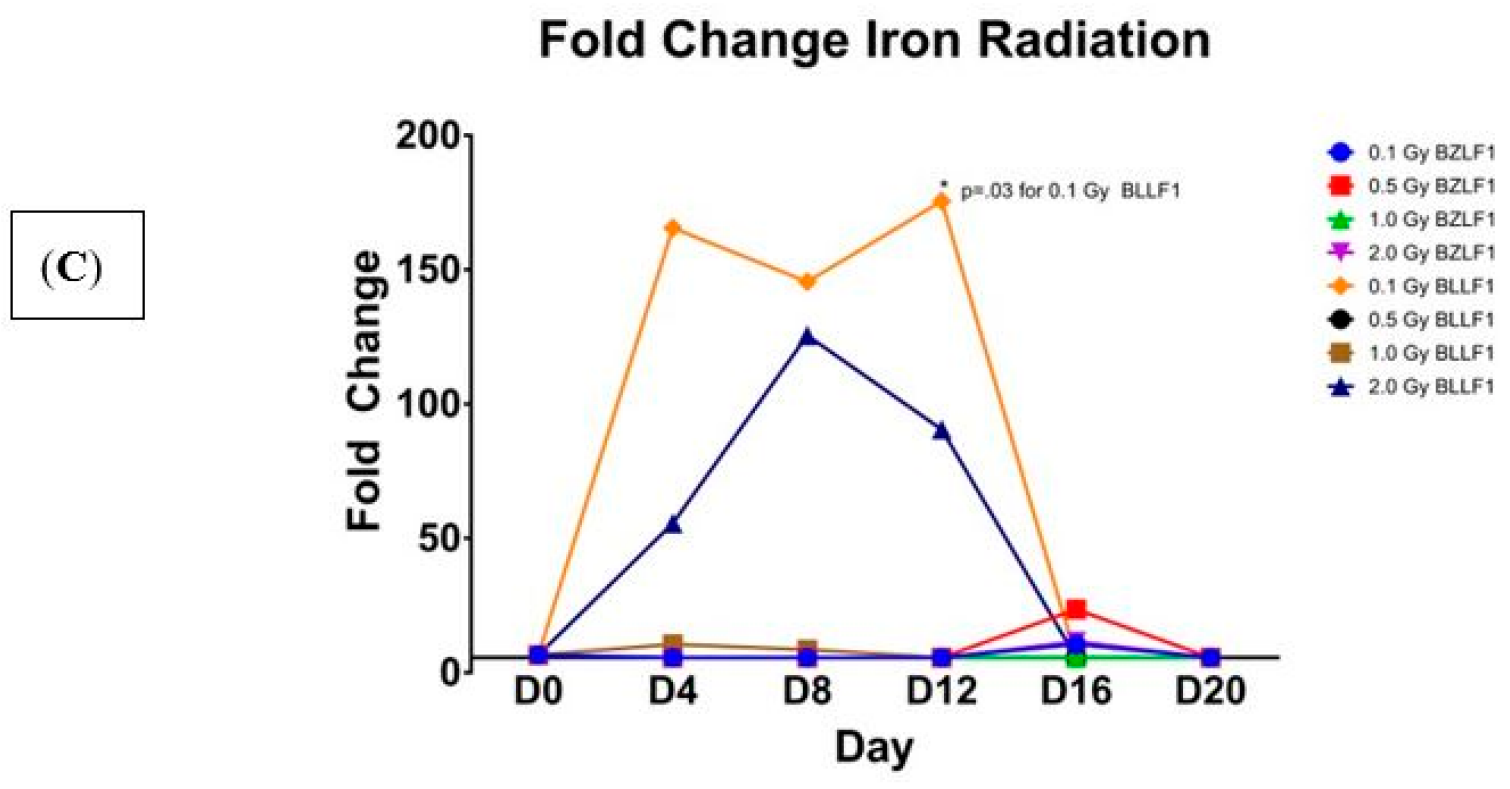
Fold change in EBV BZLF1 and BLLF1 transcripts over time at various amounts of the three types of radiation, relative to the un-irradiated control. The x axis shows the time point, measured in days, -post-radiation treatment. The y axis shows the fold change in BZLF1 and BLLF1 transcripts. BZLF1 and BLLF1 transcript quantity values divided by the corresponding GAPDH quantity values, with fold change being relative to the un-irradiated controls. Statistical significance as compared to no radiation control (two-tailed *t* test, * *p* < 0.05). (**A**) Proton; (**B**) carbon, (**C**) Iron.

We next sought to examine the effects of proton and high-energy particle radiations on their ability to induce EBV transcription (as described in detail in the Materials and Methods sections). Flasks of Akata cells were irradiated with high energy proton, carbon, and iron high energy particles. As shown in Figure 2, all three of these radiations induced transcription of BZLF1 and BLLF1. Interestingly, the high-energy proton induced the greatest gene expression of BZLF1 at the 0.1 Gy value at day 4 (Figure 2A), whereas carbon radiation induced the greatest BZLF1 transcription at 0.5 Gy, but this occurred much later, at 16 days post-radiation treatment (Figure 2B). Similar to the case for carbon, iron radiation increased BZLF1 expression at day 12 at 0.1, 0.5, and 1.0 Gy (Figure 2C).

Further analyses reveal that the values for transcription activation for protons at 0.1 Gy is significantly different than 0.5, 1.0, and 2.0 Gy at day 4 (Figure 2A). Additionally, at day 4, the value for protons at 0.1 Gy is significantly different than 1.0 Gy and 2.0 Gy. This suggests that a lower dose of radiation is responsible for inducing a greater amount of BZLF1 transcription. For BLLF1 following proton treatment at 1.0 Gy, transcription is significantly different than 0.1 Gy and 0.5 Gy at day 4 for normalized BLLF1, although the actual transcript value is much less than the values for proton irradiation. This suggests that a larger dose of radiation may be required to induce significant transcription of late genes. Carbon ions appears to induce transcription of BZLF1 at days 8 and 12 post-treatment with 1.0 Gy (Figure 2B). For BLLF1, the carbon radiation induced significant transcription at 0.1 Gy at days 8 and 20, with all radiation doses inducing greater transcript values at day 12. Iron ions induced a noticeable peak in transcript values around day 12 for BZLF1, yet none are statistically significant at the *p* = 0.05 level. When iron irradiation effect on BLLF1 transcription was examined (Figure 2C), there was a wider range of transcript values, with transcription being induced as early as day 4 and continuing to day 12 at some dosages. These data indicate that all forms of radiation tested induced EBV transcription as early as four days post-treatment; however, the effects differ from gamma irradiation in the magnitude of transcript induction as well as the duration of gene activation. Like gamma radiation, high-energy particle radiation induces EBV transcription as early as day 4 post-treatment but suggest that the type of radiation impacts the peak expression of EBV transcription, with protons peaking around day 4 and carbon and iron peaking around day 12. Overall, all of the carbon and iron ions had higher relative levels of gene expression for both the intermediate early and late genes than did either gamma or proton radiation treatments.

### 2.1. Viral Load

**Figure 3:**
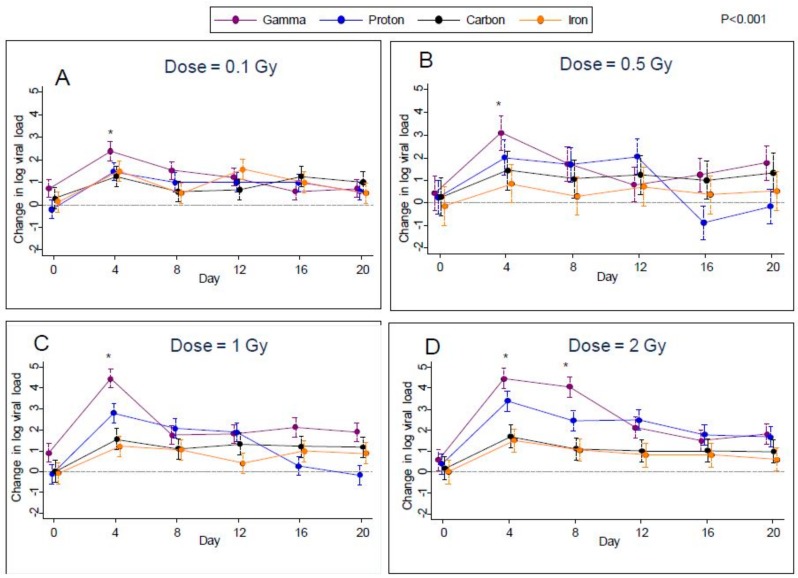
Change in viral loads (EBV DNA) compared to control following gamma, proton, carbon, and iron radiation at different dosages, (**A**); 0.1, (**B**); 0.5, (**C**), 1.0 and (**D**), 2.0 Gy treatments with 95% confidence limits indicated.

Given the increases in viral transcription, we next sought to determine if these radiation treatments induced increases in EBV genomes in the Akata cells, as evidence of productive DNA replication and viral reactivation. Plots over time of the estimated difference from control in median log viral load for all types of radiation are shown in Figure 3A–D for each of the four doses. Error bars in these plots indicate 95% confidence limits as obtained through quantile regression. From these plots, it can be seen that after irradiation, median viral loads were noticeably higher than controls at all post-irradiation time points (days 4, 8, 12, 16, and 20) for all types of radiation. More specifically, after controlling the false-discovery rate to 1%, we found significant increases in median log viral load relative to control in 67 of the possible 96 comparisons (6 time points versus 4 doses × 4 radiation types). The greatest differences were found on day 4 for all four types of radiation at all doses studied. This effect then decreased over the subsequent time points (days 8, 12, 16, and 20), except for the 2 Gy dose, where viral load levels were approximately maintained up to day 8.

For the first eight days post-irradiation, gamma radiation produced the highest rates of viral reactivation; however, on day 0 samples from the flasks designated for subsequent gamma radiation at dosages of 0.1 and 1.0 Gy had significantly higher median viral reactivation than controls (Figure 3A–D. Therefore, it is possible that some of the increased viral reactivation in gamma-irradiated samples was not actually due to radiation. However, no such effect was noted for the gamma-irradiated samples designated for irradiation at 0.5 Gy and 2.0 Gy.

### 2.2. Cell Viability

**Figure 4:**
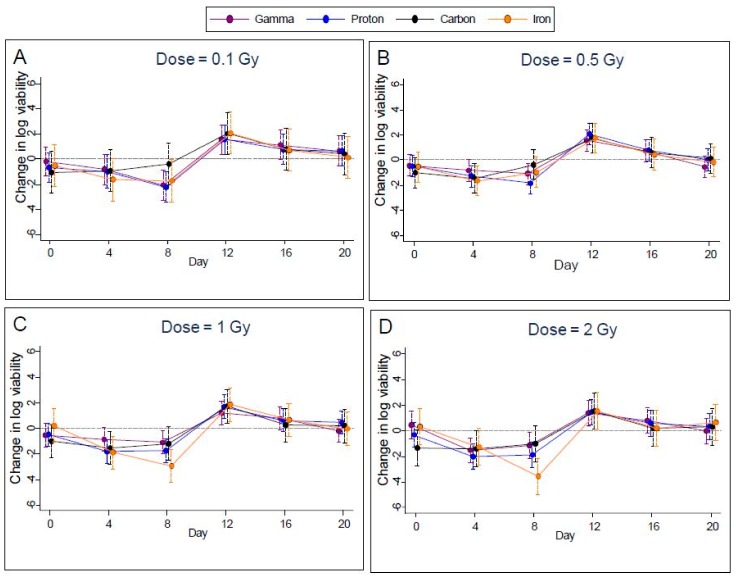
Change in cell viability compared to controls following gamma, proton, carbon, and iron radiation treatments at different dosages (**A**); 0.1, (**B**); 0.5, (**C**), 1.0 and (**D**), 2.0 Gy with 95% confidence limits indicated.

Measured cell viability of EBV infected Akata cells was highly variable and exhibited approximately the same trend in time for all radiation types (Figure 4A–D) for 0.1, 0.5, 1.0, and 2.0 Gy). With FDR control to 1%, the *p*-value threshold for identifying significant differences from controls was 0.0014. Using this threshold, we found significantly lower viability of these cells on day 8 in seven cases (gamma rays at 0.1 Gy, protons at 0.1, 0.5 1.0, and 2.0 Gy, and iron ions at 1.0 and 2.0 Gy), and on day 4 in two cases (protons at 1.0 and 2.0 Gy). Carbon did not show any significant change in the cell viability at any of the doses tested and at any of the time points in the study. In addition, viability rebounded to exceed that of controls for three cases (gamma rays at 0.5 Gy and protons at 0.5 Gy and 1.0 Gy) on day 12. After day 12, viability declined to approach values seen on day 0, see Figure 4(A–D) for the dosage of 0.5 Gy. Plots for other doses were similar.

### 2.3. Cell Size

**Figure 5:**
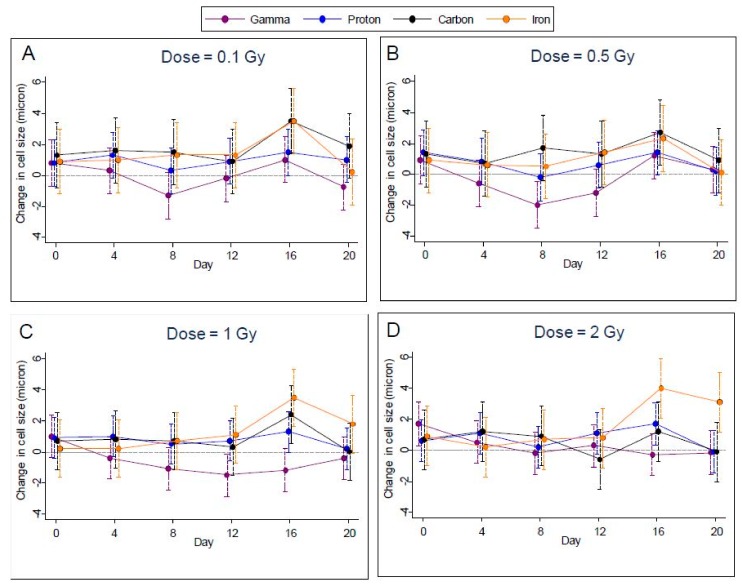
Change in cell size compared to controls following gamma, proton, carbon, and iron radiation treatments at different dosages (**A**); 0.1, (**B**); 0.5, (**C**), 1.0 and (**D**), 2.0 Gy with 95% confidence limits indicated.

Measurements of cell size were highly variable, making it difficult to discern any effect of radiation for specific dosages and days. For gamma-irradiated samples, cell size was reduced relative to controls for all but the highest dosage, while for the other types of radiation cell sizes were about the same as controls (Figure 5A–D); however, after controlling the FDR to 1%, we were able to identify only one case of a significant increase within radiation types—for iron radiation at the highest dosage on day 16. Accordingly, we noted a significant difference (*p* < 0.0002) between the radiation types on day 16 at the highest dosage.

### 2.4. Supplementary Materials

Tables of estimates, with standard errors and *P* values, for all comparisons with controls for viral reactivation, cell viability, and cell size are included in the Supplementary Materials. 

#### Simulation of Irradiated Volume

**Figure 6:**
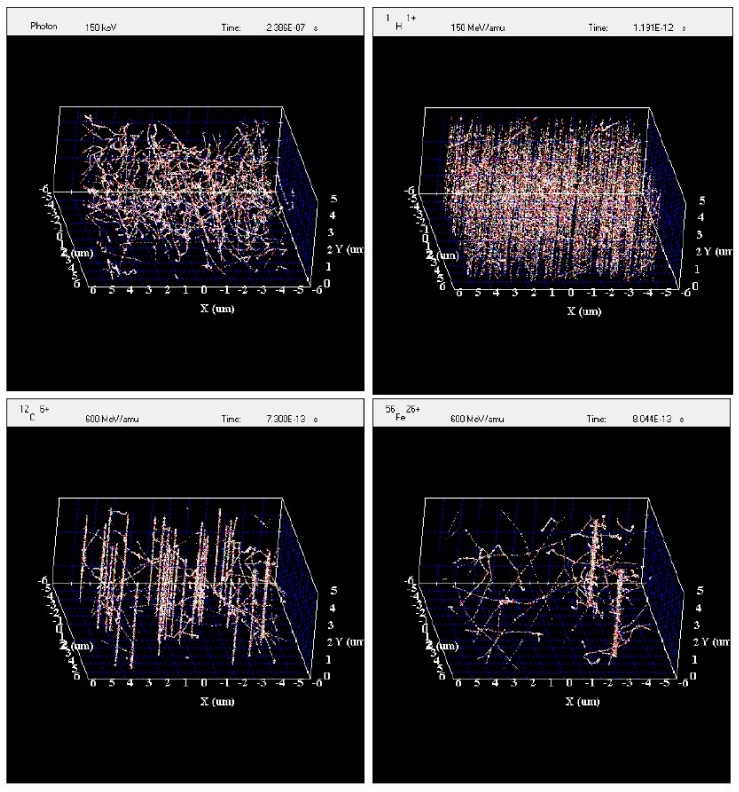
Simulation of the irradiation of a volume by ^137^Cs photons, 150-MeV protons, 600 MeV/n carbon ions, and 600 MeV/n iron ions. In all cases, the dosage to the irradiated volume is ~0.5 Gy.

The experimental data show that viral reactivation is dependent on the type of radiation used. At the scale of the Akata cells, the track structures of the radiation used for this study differ greatly from one type to another. It might be useful to use the radiation track structure to understand the differences between radiation types in viral reactivation. The simulation of an irradiated volume of 10 µm × 10 µm × 5 µm by gamma rays, protons, carbon ions, and iron ions by RITRACKS is shown in Figure 6. This volume is roughly the size of one Akata cell. The dose to the volume is approximately 0.5 Gy in all cases. This dose was chosen to better illustrate the difference between irradiation by gamma rays and ions. In Figure 6 on the top left, electron tracks corresponding to Compton and photoelectron effects are observed, and on the top right 575 proton tracks are shown. The LET of the proton used for the simulation was 0.3 keV/μm. Therefore, this is often considered sparsely ionizing radiation (as opposed to high-LET radiations). However, the energy distribution and spatial configuration of the tracks are quite different for the same dose of gamma rays and protons. Tracks from higher LET ions are also shown. On the bottom left, 34 tracks from 600 MeV/n carbon ions are shown. On the bottom right, only two tracks for 600-MeV/n iron ions are sufficient to yield a dose of 0.5 Gy.

## 3. Discussion

We studied the effect of four different types of radiation (^137^Cs gamma rays, 150-MeV protons, 600 MeV/n carbon ions, and 600 MeV/n iron ions) on induction of lytic gene transcription and reactivation of EBV in a latently infected cell line, their cell size and viability at doses of 0.1, 0.5, 1.0, and 2.0 Gy. The doses are physical doses. RBE of 1.1 for protons is referred to other biological endpoints, such as chromosome aberrations or survival, but not for the endpoint of virus activation. The changes in viral load are presented without any corrections. However, by looking at the graphs of the viral reactivation data, a correction by a factor of 1.1 on either the dosage or change in viral load would clearly be insufficient to explain the difference between the protons and gammas. The greatest differences were found on day 4 for all four types of radiation at all doses studied. This effect then decreased over the subsequent time points (days 8, 12, 16, and 20) except for 2 Gy dose where viral load levels were approximately maintained up to day 8. Gamma radiation generally produced the highest rates of viral reactivation when compared to controls followed by protons, carbon ions, and iron ions at all the doses and at all the time points. When the initiation of viral transcription was examined, a prerequisite for EBV DNA replication and reactivation, some interesting trends emerged. Gamma irradiation induced transcription of both the immediate early and late genes, peaking at day 4. In contrast, the high energy particles, while also inducing transcription initially at day 4, in many instances resulted in a second peak of transcription at later times (day 14 and 18). While the mechanism of this second round of transcription is not clear, it could be speculated that it is the result of secondary effects of the initial radiation exposure on the cell, which is translated to the viral genome again at a later time point. However, there is good correspondence between the initial increase in EBV lytic gene transcription at day 4 with increases in EBV genome load. It should be noted though, that initiation of immediate early (IE) gene transcription of the herpesviruses (in this case BZLF1 of EBV) does not necessarily represent progression to reactivation. There are many examples of initiation of IE gene activation that are abortive and do not proceed to productive reactivation. It is for this reason that we also examined a late gene (BLLF1) as well as analyzed changes in amounts of EBV genomes in order to determine whether initial transcriptional activation of an IE gene, followed by a late gene resulted in an increase in EBV genomes, likely reflecting productive reactivation. Considering this, we present strong evidence that all forms of radiation examined both resulted in initiation of E and L gene transcription and in increase in viral genomes at four days post-treatment. Therefore, we feel confident that this reflects productive reactivation at this time point. In contrast, the transcriptional events that we detected at later time points (day 12) did not coincide with a statistically significant increase in viral genomes. Therefore it is unclear whether this transcription reflected abortive reactivation, or if productive reactivation occurred, this happening in only a small number of cells that we were not able to detect. Further studies will be required to determine the mechanism and biological consequences of this second late peak in transcription.

Gamma rays and protons are usually considered much alike in radiobiology in terms of cell survival and chromosome aberrations; however, we observed that viral reactivation was induced differently. At this point it is not possible to formally identify the cause of the differences in viral reactivation resulting from proton and gamma irradiation because many factors may be involved, like damage to DNA and/or other biomolecules such as proteins and enzymes, which may be dependent on the type of radiation. The radiation track structure simulations clearly illustrate that for the same dose of gamma rays and protons, the electron tracks are quite different resulting in a different pattern of energy deposition. It would be interesting to try to link simulation results, such as DNA damage induction, with viral reactivation data in future work to investigate how the radiation track structure affects the viral reactivation. 

Because we were an exploratory study, it was necessary to use the conservative approach of controlling the false discovery rate to 1% in reporting significant effects. For this reason, differences that appear beyond chance variation when taken in isolation were not reported as being significant here. On the other hand, we feel confident that those effects we did flag as significant have an excellent chance of being reproducible.

When travelling to Mars, it is estimated that every cell nucleus in an astronaut’s body would be hit by a proton or secondary electron (e.g., electrons of the target atoms ionized by the HZE ion) every few days and by an HZE ion about once a month [3]. Therefore, space radiation is expected to be a very important stressor for astronauts, which may have direct effects on the reactivation of latent viruses that are likely exacerbated by the state of decreased immunity. During spaceflight the immune system is altered, e.g., decreased cellular immunity has been observed in Space Shuttle astronauts after only a few days in orbit [14]. Furthermore, recent studies have shown dysregulation of T-cell function and cytokine production profiles during long-duration spaceflight aboard the International Space Station [15,16]. In this in vitro experiment the immune system was not present thereby providing strong evidence that radiation of latently infected cells alone is likely a major contributor to the reactivation of herpes viruses observed in astronauts.

Space radiation is composed of a large number of different types of ions with various energies. There is also the possibility of nontargeted effects in an irradiated tissue. Because the role of ionizing radiation on virus reactivation is not well understood, more research is needed to understand the mechanisms of radiation-induced virus reactivation and the effects of dose, dose-rate, ion types, and energies. Another interesting result of our studies is that radiation-induced viral reactivation occurs within days whereas radiation carcinogenesis usually takes months or years to develop. Understanding the molecular events involved in viral reactivation will provide important new insights to radiation-induced human diseases associated with EBV. 

Our study has also shown that virus reactivation may be an adverse consequence of radiation exposure not necessarily related to spaceflight radiation, but also to other sources such as radiotherapy treatment. The radiation exposure could lead to reactivation of viruses other than EBV, such as VZV resulting in shingles. For example, a recent study has shown that, compared with incidence rates of shingles reported in a general U.S. population, the age- and sex-standardized rates of shingles were 4.8 times higher (95% confidence interval (CI), 4.0–5.6) in patients with hematologic malignancies and 1.9 times higher (95% CI, 1.7–2.1) in those with solid tumors (*23*). It is not clear, however, whether radiotherapy directly reactivates virus in latently infected cells or simply suppresses the immune system to allow viral reactivation to proceed. Thus, a better understanding of radiation-induced reactivation of EBV and other herpes viruses should provide important knowledge for spaceflight as well as terrestrial medicine.

In conclusion, four types of radiation (^137^Cs gamma rays, 150-MeV protons, 600 MeV/n carbon ions, and 600 MeV/n iron ions) induced EBV lytic gene transcription and reactivation of EBV in a latently infected cell line at doses of 0.1, 0.5, 1.0, and 2.0 Gy. This was observed in the absence of any cytokine production or involvement of the immune system. Our finding that gamma rays are more effective than protons, carbon ions, and iron ions in inducing latent virus reactivation challenges the RBE value of 1.1, which is used for protons, and also shows that RBE may be smaller than 1 for carbon ions and iron ions.

## 4. Materials and Methods

The current study was approved and sponsored under Research Opportunities in Space Biology Solicitation: NNH12ZTT002N: Proposal Number: 12-12GB_Step2-0014.

### 4.1. Cell Cultures

An Epstein‒Barr virus (EBV) producer line, designated Akata (ATCC), was established from a Japanese patient with Burkitt’s lymphoma and used in this study [12]. Akata cells have a t(8q−; 14q+) chromosomal translocation—typical of Burkitt’s lymphoma cells. These cells were cultured in RPMI 1640 Medium (Gibco^®^, Carlsbad, CA, USA) with 10% Fetal Bovine Serum (FBS) (ATCC^®^ 30-2020^™^, ATCC, Manassas, VA, USA), 25 U/mL penicillin, 25 µg/mL Streptomycin, and 2 mM L-Glutamine (Gibco^®^, Carlsbad, CA, USA) [17]. Cultures were incubated at 37 °C with 95% humidity and 5% carbon dioxide.

### 4.2. Experimental Conditions

For all experiments, 102 upright, 75-cm^2^ (T-75) tissue-culture flasks with vented caps (Corning Inc., Corning, NY, USA) were initially seeded with 1 × 10^6^ Akata cells/mL in 50 mL of fresh medium on day 0. Six flasks of these virus-infected cells were assigned to a control arm, while the remainder were split evenly (24 apiece) between four radiation exposure arms (^137^Cs gamma rays, protons, carbon ions, and iron ions). After seeding on day 0, the 24 flasks in each radiation type were divided into four irradiation groups of 6 flasks each. Depending on the group, cells were then exposed to an acute dose of either 0.1, 0.5, 1.0, or 2.0 Gy at room temperature. The dose rates in cGY/min for gamma rays, proton ions, carbon ions, and iron ions are shown in Table 1. The six flasks in the controls were not irradiated. Doses of radiation of 5 Gy or more were not studied because all cells were killed at these doses. For all four radiation types, cells were allowed to grow for up to 20 days after each exposure (day 0).

Cells were aseptically split and harvested from each flask by removing 25 mL of cells and media on day 0, and then on every fourth day for 20 days. In each of the experiments, the first harvest (day 0) was made before irradiation, while all other samples were harvested post-irradiation. The control samples were not irradiated. Except for day 20 (the last day), harvested cells and media were replaced with 25 mL of fresh RPMI 1640 Medium. After each harvest, live cells were split into five aliquots of 1 × 10^6^ cells/mL and then frozen for later assessment of cell size, cell viability, detection of EBV lytic RNA transcripts, and EBV viral load. All measurements were conducted in quadruplicates.

Proton, carbon, and iron irradiation experiments were performed at the NASA Space Radiation Laboratory at the Brookhaven National Laboratory’s facilities in New York. ^137^Cesium gamma irradiation was performed at both the NASA Johnson Space Center and the Brookhaven National Laboratory. The energy of protons was 150 MeV/n, of carbon ions was 600 MeV/n, and of iron ions was 600 MeV/n. 

### 4.3. Cell Counts and Viability

Cell count, concentration, diameter, and percentage viability of the fresh cells were measured before freezing (Cellometer Auto 2000, Nexcelom, Lawrence, MA, USA) in 1 million EBV-positive Akata cells in each condition. Cellometer Auto 2000 gives a clear distribution of live and dead cells when counted. Only live cell counts were accounted for when splitting the cells. 

### 4.4. Analysis of EBV Lytic Gene Transcription

Irradiated and control samples were harvested into RNALater™ (Thermo Fisher, Waltham, MA, USA) and stored at −80 °C until extraction. Cells were pelleted by centrifugation at 4 °C, the RNALater™ removed, 1 mL TRIzol™ Reagent (Thermo Fisher) was added to the pellets, and the pellets homogenized. Chloroform (0.2 mL) was added, the tubes vortexed vigorously, incubated for 3 min at room temperature, and centrifuged for 15 min at 12,000× *g* at 4 °C. The aqueous layer containing the RNA was removed and the RNA was precipitated by adding 0.5 mL 100% isopropanol and 0.5 µL GlycoBlue™ (Thermo Fisher). RNA was pelleted by centrifugation at 12,000× *g* for 10 min at 4 °C, the pellets were washed three times with 1 mL of 75% ethanol, then vortexed and centrifuged at 7500× *g* for 5 min at 4 °C between each wash. The pellets were air-dried and resuspended in 45 µL of nuclease free water. The RNAs were prepared for reversed transcription using the TURBO DNA-free™ DNase Kit (Ambion, Austin, TX, USA) in accordance with the manufacturer’s instructions, and the final concentration of RNA determined using a NanoDrop™ spectrophotometer. The RNA was reverse-transcribed to cDNA using the Omniscript^®^ Reverse Transcription Kit (Qiagen, Hilden, Germany) with 1 µg RNA and random decamer primers according to the manufacturer’s instruction. For quantification of the cDNAs, qPCR was performed using TaqMan™ Fast Universal Master Mix and the appropriate primers/probes (Applied Biosystems) in a StepOnePlus™ qPCR instrument. The sequences of the primers and probes used were: GAPDH Assay No. Hs02786624_g1 (Applied Biosystems), and Applied Biosystems custom designed BZLF1 forward primer: 5′AAATTTAAGAGATCCTCGTGTAAAACATC3′, reverse primer: 5′CGCCTCCTGTTGAAGCAGAT3′, probe: 5′ATAATGGAGTCAACATCCAGGCTTGGGC3′; BLLF1 forward primer: 5′AGAATCTGGGCTGGGACGTT3′, reverse primer: 5′ACATGGAGCCCGGACAAGT3′, and probe: 5′AGCCCACCACAGATTACGGCGGT3′. Assays, either radiation treatment and no radiation treatment (control), were performed on at least three biological replicates of each treatment and three technical replicates of each cDNA sample. The mean quantity for BLLF1 and BZLF1 qRT-PCR values was divided by the corresponding cellular control (GAPDH) qRT-PCR mean values for a given sample. For example, the mean value for the 0.1 Gy iron, day 0, BLLF1 qRT-PCRs was divided by the mean values for the 0.1 Gy iron, day 0, GAPDH qRT-PCRs to yield the relative quantity value (normalized to the cellular control). Once that normalization was complete, each sample was divided by its corresponding day-0 value to yield a fold change from the day-0 time point. For example, the 0.5 Gy carbon, day 8, BZLF1/GAPDH value was divided by the 0.5 Gy carbon, day 0, BZLF1/GAPDH value to yield a fold change in normalized mean quantity value between day 0 and day 8 for this sample. 

### 4.5. Determination of EBV Load

One million EBV-infected Akata cells were separated from the supernatant by centrifugation at 2500 rpm for 10 min. DNA was extracted from both cells and supernatant using an ArchivePure DNA Tissue kit (5 Prime, Gaithersburg, MD, USA). EBV BALF5 DNA was quantified by Real Time PCR (RT-PCR) using a TaqMan™ 7900HT Fast Real-time PCR System (Applied Biosystems, Grand Island, NY, USA). The EBV viral load assay used the primers and probe set as described [18] at a concentration of 150 nM and 200 nM, respectively. The results were normalized to the total DNA concentration obtained via a NanoDrop™ 1000 Spectrophotometer (Thermo Scientific, Waltham, MA, USA). For determining viral load, qPCR was performed in 20 uL reaction mixture containing absolute fast qPCR master mix using the Fast Reaction plates on the TaqMan™ 7900HT Fast Real-Time PCR System (Applied Biosystems, Grand Island, NY, USA). A fluorogenic probe, 5′/6-FAM-TGTACACGCACGAGAAATGCGCC-TAMRA/3′, was synthesized from Integrated DNA Technologies. The primers, 5′CGGAAGCCCTCTCTGGACTTC-3′ and 5′CCCTGTTTATCCGATGGAATG-3′, from the BALF5 gene were also synthesized from IDT DNA Technologies, Inc. (Coralville, IA, USA). The optimized qPCR reaction mix contained 10% of DNA, 1× TaqMan™ Master Mix, 200 µM each primer, 150 µM probe, and 43.5% water. Amplification conditions consisted of denaturation at 95 °C for 10 min, followed by 40 cycles of 15 s at 95 °C and 1 min at 60 °C. Florescence from probe degradation was recorded during the 60 °C extension cycle and analyzed by SDS program (Sequence Detector Software; Applied Biosystems). The SDS determined cycle threshold (CT) is defined as the cycle number at which the florescence value intersects the threshold value, which was set 10-fold above the background fluorescence and was within the logarithmic phase of PCR amplification. A standard curve was obtained using a plasmid containing the BALF5 gene as a standard that was serially diluted, and used to calculate the viral copy number for each reaction well that was run. The standards included in the qPCR assay yielded an inverse relationship between CT values and the amount of template DNA.

### 4.6. Normalizing Data from qPCR

For each sample, viral copy numbers from four readings were averaged and then normalized by the DNA concentration determined by NanoDrop™ to produce the main outcome measure of viral load: copies of EBV DNA per nanogram of DNA. 

### 4.7. Data Analysis

Statistical analyses of EBV lytic gene transcription data for the Akata cells were performed using the RM two-Way ANOVA Matched Values and Tukey’s Multiple Comparisons Test on GraphPad Prism to determine the significance of these values as compared to other levels of each type of radiation. EBV viral load, viability, and cell size as a function of radiation type, dosage, and day of sample were assessed with quantile regression. Quantile regression was the analysis method of choice for these data because it can accommodate skewness and high variability in the data without assuming any particular statistical distribution. Dependent variables for the regressions were log viral load, logit viability, and cell size (untransformed). For each of these, a separate regression was run for each dose (0.1, 0.5, 1.0, and 2.0 Gy), because dose‒response patterns were generally inconsistent over the various combinations of radiation category and time. Also, in the regression models, time points were treated as discrete conditions because there was an insufficient number or density of these time points immediately after irradiation of samples to formulate continuous time-trend models. This was an exploratory study and, as such, we were seeking to identify combinations of dosages, days, and radiation types that produced median responses sufficiently different from the corresponding control samples to be beyond chance variation. Taking into consideration the 96 possible comparisons (4 radiation types × 4 doses × 6 days) that could be made for each outcome, we used the method of Benjamini and Yekutieli [19] to control the false-discovery rate (FDR) for flagging combinations of interest so that the expected number of false discoveries would be one or less for each outcome variable (see results). Although theoretically there should not be any differences on day 0 (before irradiation) we included all the day-0 comparisons as a check for consistency of samples. 

### 4.8. Simulations of Radiation Tracks

To understand the differences in the reactivation of virus by radiation types, it is useful to look at the radiation track structure of the radiation types used in this study. For heavy ions, energy deposition is highly heterogeneous, with a localized contribution along the trajectory of every particle and lateral diffusion of energetic electrons (i.e., gamma rays, the target atom electrons ionized by the incident heavy ion and emitted at high energy) many microns from the path of the ions. These particles are therefore densely ionizing along the primary track (e.g., the track followed by the incident heavy ion, the so-called core). Moreover, they are surrounded by a region (penumbra) comprising the high-energy electrons ejected by ions [20]. The density of the core and penumbra depends mostly on the charge and velocity (energy per nucleon) of the ion. The situation is quite different for gamma rays that interact with the medium mostly by the Compton and photoelectric effects, which result in considerable deflection of the photon after an interaction and the creation of a large number of electron tracks in the medium. 

In this work, we have simulated the irradiation of a representative volume (10 µm × 10 µm × 5 µm) by gamma rays, protons, carbon and Iron ions using the code RITRACKS (Relativistic Ion Tracks). The code RITRACKS, developed over the last few years at the NASA Johnson Space Center [21], simulates the primary interactions of the radiation and calculates the energy of all electrons produced in the medium. The secondary electrons may lead to further ionizations in the medium, and secondary electron tracks are simulated as well. The detailed algorithms for these simulations have been described [22,23]. Periodic boundary conditions are applied to mimic the contributions of radiation from neighboring volumes to the volume of interest.

## Figures and Tables

**Table 1:** Dosage rate (cGy/min) for different radiation types.

Type	LET (keV/µm)	0.1 Gy	0.5 Gy	1.0 Gy	2.0 Gy
Gamma Rays	0.30	148.76	148.76	148.76	148.76
Protons 150 MeV	0.54	9.71	44.64	49.76	55.71
Carbon Ions 600 MeV/n	9.18	18.87	29.24	27.55	27.55
Iron Ions 600 MeV/n	172.40	20.00	14.71	38.46	23.53

## Supplementary Materials

Supplementary materials can be found at http://www.mdpi.com/1422-0067/19/10/2961/s1.
