# Supplementary material for: Reactivation of Latent Epstein-Barr Virus: A Comparison after Exposure to Gamma, Proton, Carbon, and Iron Radiation
Source: Int J Mol Sci. 2018 Sep 28;19(10):2961. doi: 10.3390/ijms19102961 (PMC6213004; doi:10.3390/ijms19102961)
Supplement: Supplementary file 1 [file ijms-19-02961-s001.pdf]

Supplementary Material for Statistical Analysis

Viral Reactivation							
Radiation	dose	day	Estimated DIfference from Control*	standard error	lower 95% conf	upper 95% conf	p-value
Gamma	0.1	0	0.727	0.207	0.317	1.137	0.000623
Gamma	0.1	4	2.380	0.207	1.970	2.790	2.40E-21
Gamma	0.1	8	1.522	0.207	1.112	1.932	2.23E-11
Gamma	0.1	12	1.228	0.207	0.818	1.638	2.72E-08
Gamma	0.1	16	0.595	0.207	0.185	1.005	0.004754
Gamma	0.1	20	0.731	0.207	0.321	1.141	0.000584
Gamma	0.5	0	0.412	0.385	-0.350	1.173	0.286982
Gamma	0.5	4	3.084	0.385	2.322	3.846	6.51E-13
Gamma	0.5	8	1.714	0.385	0.952	2.475	1.85E-05
Gamma	0.5	12	0.788	0.385	0.027	1.550	0.042597
Gamma	0.5	16	1.236	0.385	0.475	1.998	0.001673
Gamma	0.5	20	1.766	0.385	1.004	2.528	1.07E-05
Gamma	1	0	0.894	0.230	0.440	1.349	0.00016
Gamma	1	4	4.453	0.230	3.999	4.908	0
Gamma	1	8	1.746	0.230	1.291	2.201	5.96E-12
Gamma	1	12	1.799	0.230	1.345	2.254	1.72E-12
Gamma	1	16	2.129	0.230	1.674	2.583	6.78E-16
Gamma	1	20	1.903	0.230	1.449	2.358	1.51E-13
Gamma	2	0	0.578	0.250	0.082	1.074	0.022669
Gamma	2	4	4.465	0.250	3.969	4.960	3.02E-36
Gamma	2	8	4.074	0.250	3.578	4.570	9.68E-33
Gamma	2	12	2.119	0.250	1.623	2.615	5.75E-14
Gamma	2	16	1.493	0.250	0.997	1.988	2.37E-08
Gamma	2	20	1.802	0.250	1.306	2.298	4.97E-11
Proton	0.1	0	-0.215	0.207	-0.625	0.195	0.301536
Proton	0.1	4	1.479	0.207	1.069	1.888	6.63E-11
Proton	0.1	8	0.993	0.207	0.583	1.403	4.53E-06
Proton	0.1	12	0.996	0.207	0.586	1.406	4.25E-06
Proton	0.1	16	0.985	0.207	0.575	1.395	5.31E-06
Proton	0.1	20	0.605	0.207	0.195	1.015	0.004152
Proton	0.5	0	0.243	0.385	-0.519	1.005	0.529046
Proton	0.5	4	2.002	0.385	1.240	2.763	7.82E-07
Proton	0.5	8	1.684	0.385	0.922	2.446	2.52E-05
Proton	0.5	12	2.042	0.385	1.280	2.804	4.89E-07
Proton	0.5	16	-0.889	0.385	-1.651	-0.128	0.02248
Proton	0.5	20	-0.167	0.385	-0.929	0.595	0.665027
Proton	1	0	-0.137	0.230	-0.592	0.317	0.551194
Proton	1	4	2.795	0.230	2.340	3.250	5.41E-23
Proton	1	8	2.066	0.230	1.611	2.521	3.08E-15
Proton	1	12	1.875	0.230	1.421	2.330	2.91E-13

Proton	1	16	0.254	0.230	-0.201	0.709	0.270755
Proton	1	20	-0.185	0.230	-0.640	0.269	0.421268
Proton	2	0	0.385	0.250	-0.110	0.881	0.126443
Proton	2	4	3.399	0.250	2.904	3.895	2.07E-26
Proton	2	8	2.465	0.250	1.969	2.961	2.68E-17
Proton	2	12	2.494	0.250	1.998	2.990	1.40E-17
Proton	2	16	1.784	0.250	1.288	2.279	7.28E-11
Proton	2	20	1.678	0.250	1.182	2.174	6.29E-10
Carbon	0.1	0	0.283	0.232	-0.176	0.741	0.224656
Carbon	0.1	4	1.264	0.232	0.806	1.722	2.45E-07
Carbon	0.1	8	0.585	0.232	0.127	1.044	0.012711
Carbon	0.1	12	0.662	0.232	0.203	1.120	0.005005
Carbon	0.1	16	1.260	0.232	0.801	1.718	2.68E-07
Carbon	0.1	20	0.996	0.232	0.538	1.454	3.37E-05
Carbon	0.5	0	0.254	0.430	-0.598	1.106	0.555907
Carbon	0.5	4	1.423	0.430	0.571	2.275	0.001231
Carbon	0.5	8	1.055	0.430	0.203	1.906	0.01563
Carbon	0.5	12	1.233	0.430	0.381	2.084	0.004893
Carbon	0.5	16	0.992	0.430	0.141	1.844	0.022767
Carbon	0.5	20	1.320	0.430	0.469	2.172	0.002638
Carbon	1	0	0.009	0.257	-0.500	0.517	0.97303
Carbon	1	4	1.551	0.257	1.043	2.060	1.62E-08
Carbon	1	8	1.088	0.257	0.580	1.597	4.36E-05
Carbon	1	12	1.319	0.257	0.810	1.827	1.05E-06
Carbon	1	16	1.199	0.257	0.690	1.707	7.74E-06
Carbon	1	20	1.168	0.257	0.660	1.676	1.26E-05
Carbon	2	0	0.169	0.280	-0.385	0.723	0.547654
Carbon	2	4	1.699	0.280	1.145	2.254	1.41E-08
Carbon	2	8	1.106	0.280	0.551	1.660	0.00013
Carbon	2	12	1.013	0.280	0.459	1.567	0.00043
Carbon	2	16	1.023	0.280	0.469	1.577	0.000378
Carbon	2	20	0.981	0.280	0.427	1.536	0.000635
Iron	0.1	0	0.135	0.232	-0.323	0.594	0.559999
Iron	0.1	4	1.489	0.232	1.031	1.948	2.38E-09
Iron	0.1	8	0.517	0.232	0.059	0.976	0.027269
Iron	0.1	12	1.572	0.232	1.114	2.030	4.00E-10
Iron	0.1	16	0.985	0.232	0.527	1.443	4.06E-05
Iron	0.1	20	0.517	0.232	0.059	0.975	0.027356
Iron	0.5	0	-0.157	0.430	-1.009	0.695	0.716015
Iron	0.5	4	0.831	0.430	-0.021	1.682	0.05583
Iron	0.5	8	0.283	0.430	-0.569	1.134	0.512238
Iron	0.5	12	0.709	0.430	-0.143	1.561	0.101956
Iron	0.5	16	0.357	0.430	-0.494	1.209	0.407759
Iron	0.5	20	0.508	0.430	-0.344	1.359	0.240282
Iron	1	0	-0.092	0.257	-0.600	0.416	0.720619
Iron	1	4	1.210	0.257	0.702	1.718	6.44E-06
Iron	1	8	1.038	0.257	0.529	1.546	9.26E-05

Iron	1	12	0.399	0.257	-0.110	0.907	0.123324
Iron	1	16	0.969	0.257	0.461	1.477	0.000248
Iron	1	20	0.875	0.257	0.367	1.384	0.00088
Iron	2	0	0.015	0.280	-0.540	0.569	0.958216
Iron	2	4	1.509	0.280	0.955	2.063	3.38E-07
Iron	2	8	1.051	0.280	0.497	1.605	0.000265
Iron	2	12	0.809	0.280	0.255	1.363	0.004546
Iron	2	16	0.812	0.280	0.258	1.366	0.004402
Iron	2	20	0.605	0.280	0.050	1.159	0.032724

* change in median log EBV DNA copies per nanogram of DNA

Cell Viability							
Radiation	dose	day	Estimated DIfference from Control*	standard error	lower 95% conf	upper 95% conf	p-value
Gamma	0.1	0	-0.188	0.593	-1.365	0.989	0.751873
Gamma	0.1	4	-0.817	0.593	-1.994	0.360	0.171702
Gamma	0.1	8	-2.057	0.593	-3.234	-0.880	0.000777
Gamma	0.1	12	1.559	0.593	0.381	2.736	0.009973
Gamma	0.1	16	1.131	0.593	-0.046	2.308	0.05947
Gamma	0.1	20	0.644	0.593	-0.534	1.821	0.280731
Gamma	0.5	0	-0.421	0.425	-1.265	0.422	0.324297
Gamma	0.5	4	-0.804	0.425	-1.648	0.039	0.06151
Gamma	0.5	8	-1.072	0.425	-1.916	-0.229	0.013248
Gamma	0.5	12	1.576	0.425	0.732	2.419	0.000344
Gamma	0.5	16	0.745	0.425	-0.098	1.589	0.082638
Gamma	0.5	20	-0.533	0.425	-1.377	0.310	0.212823
Gamma	1	0	-0.541	0.483	-1.500	0.418	0.265567
Gamma	1	4	-0.868	0.461	-1.782	0.046	0.062581
Gamma	1	8	-1.097	0.461	-2.012	-0.183	0.019133
Gamma	1	12	1.208	0.461	0.293	2.122	0.010135
Gamma	1	16	0.776	0.461	-0.138	1.691	0.09522
Gamma	1	20	-0.177	0.461	-1.092	0.737	0.701123
Gamma	2	0	0.441	0.529	-0.609	1.490	0.406769
Gamma	2	4	-1.523	0.504	-2.524	-0.522	0.003208
Gamma	2	8	-1.153	0.504	-2.153	-0.152	0.024393
Gamma	2	12	1.376	0.504	0.376	2.377	0.007504
Gamma	2	16	0.766	0.504	-0.235	1.767	0.132068
Gamma	2	20	0.008	0.504	-0.992	1.009	0.987032
Proton	0.1	0	-0.668	0.593	-1.845	0.509	0.263034
Proton	0.1	4	-0.994	0.663	-2.310	0.322	0.137164
Proton	0.1	8	-2.210	0.593	-3.387	-1.033	0.000324
Proton	0.1	12	1.550	0.593	0.373	2.727	0.010369
Proton	0.1	16	0.787	0.593	-0.391	1.964	0.187985
Proton	0.1	20	0.643	0.593	-0.534	1.821	0.280818

Proton	0.5	0	-0.469	0.425	-1.312	0.375	0.272936
Proton	0.5	4	-1.264	0.425	-2.108	-0.421	0.003678
Proton	0.5	8	-1.829	0.425	-2.673	-0.986	0.000039
Proton	0.5	12	2.094	0.425	1.251	2.938	3.27E-06
Proton	0.5	16	0.766	0.410	-0.047	1.579	0.064507
Proton	0.5	20	0.025	0.446	-0.860	0.910	0.955318
Proton	1	0	-0.487	0.461	-1.402	0.427	0.292727
Proton	1	4	-1.811	0.461	-2.725	-0.897	0.000156
Proton	1	8	-1.748	0.461	-2.663	-0.834	0.000253
Proton	1	12	1.683	0.461	0.768	2.597	0.000416
Proton	1	16	0.630	0.461	-0.284	1.544	0.174718
Proton	1	20	0.444	0.461	-0.471	1.358	0.33809
Proton	2	0	-0.321	0.504	-1.322	0.680	0.526209
Proton	2	4	-2.045	0.504	-3.046	-1.044	9.92E-05
Proton	2	8	-1.865	0.504	-2.866	-0.864	0.000355
Proton	2	12	1.461	0.504	0.460	2.461	0.004641
Proton	2	16	0.603	0.504	-0.398	1.604	0.234828
Proton	2	20	0.344	0.504	-0.657	1.345	0.496916
Carbon	0.1	0	-1.062	0.839	-2.727	0.603	0.208529
Carbon	0.1	4	-0.929	0.839	-2.594	0.736	0.270856
Carbon	0.1	8	0.992	0.839	-0.673	2.657	0.239821
Carbon	0.1	12	2.032	0.839	0.367	3.697	0.017265
Carbon	0.1	16	0.677	0.839	-0.988	2.342	0.421672
Carbon	0.1	20	0.399	0.839	-1.266	2.063	0.635857
Carbon	0.5	0	-0.999	0.601	-2.192	0.194	0.099876
Carbon	0.5	4	-1.430	0.601	-2.623	-0.237	0.019263
Carbon	0.5	8	-0.352	0.601	-1.545	0.841	0.560096
Carbon	0.5	12	1.768	0.601	0.575	2.962	0.004059
Carbon	0.5	16	-0.576	0.601	-1.769	0.617	0.340724
Carbon	0.5	20	0.147	0.601	-1.046	1.340	0.807354
Carbon	1	0	-1.333	0.652	-2.626	-0.040	0.043424
Carbon	1	4	-1.514	0.652	-2.807	-0.221	0.022207
Carbon	1	8	-1.053	0.652	-2.346	0.240	0.109305
Carbon	1	12	1.819	0.652	0.526	3.112	0.006287
Carbon	1	16	0.490	0.652	-0.803	1.783	0.453619
Carbon	1	20	0.323	0.652	-0.970	1.616	0.621133
Carbon	2	0	-1.333	0.713	-2.748	0.082	0.064596
Carbon	2	4	-1.406	0.713	-2.822	0.009	0.051418
Carbon	2	8	-1.014	0.713	-2.430	0.401	0.158199
Carbon	2	12	1.779	0.713	0.363	3.194	0.014293
Carbon	2	16	0.192	0.713	-1.224	1.607	0.788825
Carbon	2	20	0.292	0.713	-1.123	1.707	0.683418
Iron	0.1	0	-0.515	0.839	-2.179	1.150	0.541038
Iron	0.1	4	-1.496	0.839	-3.160	0.169	0.077717
Iron	0.1	8	-1.851	0.839	-3.516	-0.187	0.029644
Iron	0.1	12	2.289	0.839	0.625	3.954	0.00752
Iron	0.1	16	0.756	0.839	-0.909	2.421	0.369961

Iron	0.1	20	0.344	0.839	-1.321	2.009	0.682748
Iron	0.5	0	-0.515	0.601	-1.708	0.678	0.394189
Iron	0.5	4	-1.635	0.601	-2.828	-0.442	0.007705
Iron	0.5	8	-0.967	0.601	-2.160	0.225	0.110799
Iron	0.5	12	1.830	0.601	0.637	3.022	0.002989
Iron	0.5	16	0.449	0.601	-0.744	1.642	0.456984
Iron	0.5	20	-0.155	0.601	-1.348	1.038	0.796694
Iron	1	0	0.224	0.652	-1.069	1.517	0.732005
Iron	1	4	-1.915	0.652	-3.208	-0.622	0.004095
Iron	1	8	-2.827	0.652	-4.120	-1.534	3.42E-05
Iron	1	12	1.861	0.652	0.568	3.154	0.005222
Iron	1	16	0.667	0.652	-0.626	1.960	0.308289
Iron	1	20	-0.040	0.652	-1.333	1.253	0.950854
Iron	2	0	0.309	0.713	-1.106	1.724	0.665908
Iron	2	4	-1.536	0.713	-2.952	-0.121	0.033629
Iron	2	8	-3.561	0.713	-4.976	-2.146	2.50E-06
Iron	2	12	1.550	0.713	0.135	2.965	0.032147
Iron	2	16	0.198	0.713	-1.217	1.613	0.781687
Iron	2	20	0.644	0.713	-0.771	2.059	0.369092

* change in median logit cell viability proportion

Cell Size

Radiation	dose	day	Estimated DIfference from Control*	standard error	lower 95% conf	upper 95% conf	p-value
Gamma	0.1	0	0.799	0.753	-0.694	2.292	0.2911
Gamma	0.1	4	0.300	0.753	-1.193	1.792	0.6914
Gamma	0.1	8	-1.300	0.753	-2.793	0.193	0.0872
Gamma	0.1	12	-0.201	0.753	-1.694	1.292	0.7900
Gamma	0.1	16	1.002	0.753	-0.491	2.495	0.1862
Gamma	0.1	20	-0.755	0.753	-2.248	0.738	0.3182
Gamma	0.5	0	0.897	0.789	-0.667	2.461	0.2579
Gamma	0.5	4	-0.597	0.752	-2.088	0.895	0.4291
Gamma	0.5	8	-2.000	0.752	-3.492	-0.509	0.0091
Gamma	0.5	12	-1.202	0.752	-2.694	0.289	0.1129
Gamma	0.5	16	1.200	0.752	-0.292	2.692	0.1136
Gamma	0.5	20	0.301	0.752	-1.191	1.792	0.6899
Gamma	1	0	0.999	0.694	-0.379	2.377	0.1533
Gamma	1	4	-0.398	0.694	-1.776	0.980	0.5679
Gamma	1	8	-1.100	0.694	-2.478	0.278	0.1164
Gamma	1	12	-1.498	0.694	-2.876	-0.120	0.0335
Gamma	1	16	-1.199	0.694	-2.577	0.179	0.0873
Gamma	1	20	-0.415	0.694	-1.793	0.963	0.5512
Gamma	2	0	1.699	0.719	0.272	3.125	0.0201
Gamma	2	4	0.502	0.686	-0.858	1.862	0.4658
Gamma	2	8	-0.199	0.686	-1.560	1.161	0.7718
Gamma	2	12	0.300	0.686	-1.060	1.661	0.6623

Gamma	2	16	-0.302	0.686	-1.662	1.059	0.6611
Gamma	2	20	-0.164	0.719	-1.591	1.263	0.8199
Proton	0.1	0	0.799	0.753	-0.694	2.292	0.2910
Proton	0.1	4	1.301	0.753	-0.192	2.793	0.0870
Proton	0.1	8	0.299	0.753	-1.194	1.791	0.6925
Proton	0.1	12	0.901	0.753	-0.592	2.394	0.2340
Proton	0.1	16	1.499	0.753	0.006	2.992	0.0490
Proton	0.1	20	1.001	0.753	-0.492	2.494	0.1864
Proton	0.5	0	1.400	0.752	-0.091	2.891	0.0655
Proton	0.5	4	0.800	0.752	-0.691	2.291	0.2896
Proton	0.5	8	-0.201	0.752	-1.692	1.290	0.7894
Proton	0.5	12	0.600	0.752	-0.891	2.091	0.4263
Proton	0.5	16	1.399	0.724	-0.037	2.836	0.0561
Proton	0.5	20	0.201	0.788	-1.363	1.764	0.7997
Proton	1	0	0.900	0.662	-0.413	2.213	0.1769
Proton	1	4	1.003	0.662	-0.311	2.316	0.1329
Proton	1	8	0.502	0.662	-0.812	1.815	0.4501
Proton	1	12	0.700	0.662	-0.613	2.013	0.2926
Proton	1	16	1.299	0.662	-0.014	2.612	0.0525
Proton	1	20	0.199	0.662	-1.114	1.513	0.7640
Proton	2	0	0.599	0.686	-0.761	1.959	0.3845
Proton	2	4	1.102	0.686	-0.258	2.462	0.1112
Proton	2	8	0.198	0.686	-1.162	1.559	0.7728
Proton	2	12	1.099	0.686	-0.261	2.459	0.1121
Proton	2	16	1.700	0.686	0.340	3.060	0.0148
Proton	2	20	-0.099	0.686	-1.460	1.261	0.8852
Carbon	0.1	0	1.300	1.064	-0.811	3.411	0.2248
Carbon	0.1	4	1.599	1.064	-0.512	3.710	0.1362
Carbon	0.1	8	1.499	1.064	-0.612	3.610	0.1621
Carbon	0.1	12	0.900	1.064	-1.211	3.011	0.3997
Carbon	0.1	16	3.499	1.064	1.388	5.610	0.0014
Carbon	0.1	20	1.899	1.064	-0.212	4.011	0.0773
Carbon	0.5	0	1.301	1.063	-0.807	3.410	0.2237
Carbon	0.5	4	0.700	1.063	-1.409	2.808	0.5118
Carbon	0.5	8	1.702	1.063	-0.407	3.810	0.1125
Carbon	0.5	12	1.301	1.063	-0.808	3.409	0.2239
Carbon	0.5	16	2.698	1.063	0.590	4.807	0.0127
Carbon	0.5	20	0.900	1.063	-1.209	3.008	0.3993
Carbon	1	0	0.703	0.936	-1.155	2.561	0.4545
Carbon	1	4	0.801	0.936	-1.057	2.658	0.3945
Carbon	1	8	0.700	0.936	-1.158	2.558	0.4564
Carbon	1	12	0.301	0.936	-1.557	2.159	0.7485
Carbon	1	16	2.400	0.936	0.542	4.258	0.0119
Carbon	1	20	0.001	0.936	-1.857	1.859	0.9994
Carbon	2	0	0.702	0.969	-1.221	2.625	0.4709
Carbon	2	4	0.900	0.969	-1.023	2.823	0.3556
Carbon	2	8	0.901	0.969	-1.022	2.824	0.3546

Carbon	2	12	-0.601	0.969	-2.524	1.322	0.5368
Carbon	2	16	1.199	0.969	-0.724	3.122	0.2190
Carbon	2	20	0.000	0.969	-1.923	1.923	1.0000
Iron	0.1	0	0.898	1.065	-1.214	3.010	0.4010
Iron	0.1	4	1.001	1.065	-1.111	3.113	0.3493
Iron	0.1	8	1.302	1.065	-0.810	3.413	0.2243
Iron	0.1	12	1.301	1.065	-0.811	3.413	0.2245
Iron	0.1	16	3.499	1.065	1.387	5.610	0.0014
Iron	0.1	20	0.202	1.065	-1.910	2.314	0.8499
Iron	0.5	0	0.901	1.063	-1.208	3.010	0.3985
Iron	0.5	4	0.601	1.063	-1.508	2.710	0.5729
Iron	0.5	8	0.504	1.063	-1.605	2.613	0.6365
Iron	0.5	12	1.397	1.063	-0.712	3.506	0.1918
Iron	0.5	16	2.299	1.063	0.191	4.408	0.0329
Iron	0.5	20	0.101	1.063	-2.008	2.210	0.9246
Iron	1	0	0.200	0.936	-1.658	2.058	0.8312
Iron	1	4	0.200	0.936	-1.657	2.058	0.8310
Iron	1	8	0.700	0.936	-1.158	2.557	0.4565
Iron	1	12	0.800	0.936	-1.057	2.658	0.3945
Iron	1	16	3.499	0.936	1.641	5.357	0.0003
Iron	1	20	1.802	0.936	-0.055	3.660	0.0571
Iron	2	0	0.699	0.970	-1.225	2.623	0.4729
Iron	2	4	0.202	0.970	-1.722	2.126	0.8357
Iron	2	8	0.702	0.970	-1.222	2.626	0.4709
Iron	2	12	0.800	0.970	-1.124	2.724	0.4114
Iron	2	16	3.999	0.970	2.075	5.923	0.0001
Iron	2	20	3.100	0.970	1.176	5.024	0.0019

* change in mean cell diameter (microns)
